# Possible Vicarious Traumatization Among Psychiatric Inpatients During the Remission Phase of the COVID-19: A Single-Center Cross-Sectional Study

**DOI:** 10.3389/fpsyt.2021.677082

**Published:** 2021-08-24

**Authors:** Yage Zheng, Ling Xiao, Yonglan Huang, Qing Wang, Yinping Xie, Huiling Wang, Gaohua Wang

**Affiliations:** Mental Health Center, Renmin Hospital of Wuhan University, Wuhan, China

**Keywords:** remission period, COVID-19, epidemiology, vicarious traumatization, inpatients

## Abstract

**Background:** Far from being a clinical disease, the COVID-19 pandemic has become a threatening social event worldwide exerting long-term impacts on human beings.

**Objective:** This study was designed to determine if and to what extent psychiatric inpatients during the remission phase of the pandemic suffered from vicarious traumatization.

**Method:** Totally 266 eligible participants from psychiatric and psychological wards in a hospital were recruited during October 26th, 2020 to February 4th, 2021 to finish a self-made online questionnaire consisting of Impact of Event Scale-Revised (IES-R), Self-Rating Depression Scale (SDS), Self-Rating Anxiety Scale (SAS), Obsessive-Compulsive Inventory-Revised (OCI-R), Pittsburgh Sleep Quality Index-Revised (PSQI-R), Social Support Rating Scale (SSRS), Beck Suicide Ideation Scale (SSI), 12-Item Short-Form Health Survey (SF-12). Meanwhile, some socio-demographics and information related to the pandemic were also recorded.

**Results:** The detection rate of vicarious traumatic symptoms (VTS) was 80.83%, including 40.98% for mild ones, 25.56% for moderate ones, and 14.29% for severe ones, among whom 98.14% possessed all three phenotypes. 27.07% of the sample were considered possible vicarious traumatization (pVT). Having acquaintances infected with or died from COVID-19, worries on re-outbreak of COVID-19, a higher score of OCI-R or lower score of SF-12, and long latency of VTS were independent risk factors of pVT.

**Conclusion:** Our study showed that COVID-19 could have profound mental influences on psychiatric inpatients. It is high time we did some screening in the wards to seek for patients at risk.

## Introduction

COVID-19 disease, which was first reported more than 1 year ago, has caused considerable damage to human beings worldwide. According to the World Health Organization (WHO) (1), by February 28, 2021, COVID-19 played a role in the deaths of more than 2.5 million people, leaving many families bereaved and incomplete. Moreover, trends in new cases in the preceding 7 days have been unstable, with 2.6 million newly diagnosed cases confirmed from February 21 to February 28, 2021. Uncertainty, hopelessness, and fear of the future seem to be currently spreading among people.

The term vicarious traumatization (VT) was first established by Li et al. to describe the phenomenon in which an expert psychologist was affected by the trauma conveyed by a person living with psychiatric conditions during or after the process of psychological consultation (2). Associated studies of VT have often focused on psychiatrists (3), psychologists (4), or caregivers (5), but the connotations of VT have been extended to many other populations (6), including the general population (7). VT is similar to post traumatic stress disorder (PTSD), as these conditions sharing almost the same manifestations; the main difference may lie in the source of stress. People with VT are usually a witness of the suffering of others; that is, the stress is relatively “indirect” (2).

In terms of stress due to COVID-19, Li et al. (2) surveyed 740 nurses and members of the general public; results indicated the universality of VT, with greater VT in non-frontier nurses and the general public than in frontier nurses. Another study found that of 339 psychotherapists, low, moderate, or high levels of VT were reported by 21.3, 62.7, and 14.9% of these persons, respectively (8).

Generally, PTSD can be divided into acute and chronic forms, according to the course of the disease (9). Delayed manifestations are typical of PTSD; a meta-analysis revealed that PTSD symptoms were delayed by over 6 months from the precipitating event in one in four PTSD cases (10). Studies of trauma following 9.11 and severe acute respiratory syndrome (SARS) found that PTSD could be traced for several years after these disasters (11, 12), highlighting the chronic and intractable nature of the condition. Further, researchers have also suggested that aggravation of pre-existing psychiatric conditions might occur as an epidemic develops (13, 14); however, to date no studies of VT have been conducted in such populations.

Currently many countries are continuing to campaign against the COVID-19 epidemic; as such, it is difficult to accurately determine how many people with COVID-19-related PTSD exist worldwide. Wuhan, China, as one of the first few territories that successfully controlled the pandemic, is engaged in recovery. It is known that patients living with psychiatric conditions are relatively vulnerable and sensitive to environmental changes or stress; accordingly, we questioned whether such patients in wards would exhibit a high incidence of VT (or at least potential VT, i.e., pVT). We further asked how symptoms would present, whether there might be a relationship between VT and other kinds of psychiatric symptoms, and what factors might influence VT. Accordingly, we conducted a survey of the indirect stress on inpatients living with psychiatric conditions, 6 months after the remission phase of COVID-19 began. We proposed two hypotheses:

First, VT symptoms would be prevalent among vulnerable persons, such as psychiatric inpatients, even during the remission stage of the COVID-19 pandemic.

Second, other psychiatric symptoms, such as depression, anxiety, or obsession, would be associated with pVT.

## Materials and Methods

### Participants

Psychiatric inpatients admitted to Renmin Hospital of Wuhan University (a grade-A tertiary hospital) during October 26, 2020 to February 4, 2021 were recruited for the trial.

Inclusion criteria were: (1) inpatients from psychiatric or psychological departments of Renmin Hospital of Wuhan University, (2) non-schizophrenia as the first diagnosis, (3) age above 13 years old, and (4) with at least basic literacy.

Exclusion criteria were: (1) those who were screened out on the basis of their answer to a decoy question, (2) those who refused to take part or quit during the study, (3) those who required someone else to answer on their behalf.

An electronic questionnaire was provided to respondents via WeChat.

### Materials

#### Wenjuanxin

A web app was used to collect questionnaire data from subjects; Supplementary Material 1 provides the detailed Chinese version of the questionnaire.

#### WeChat

One of the most popular apps for social interaction in China was used, for iOS version 7.0.17–8.0.2. Supplementary Material 2 details the procedures.

#### Custom Questionnaire

A custom questionnaire containing three parts was developed.

First, socio-demographic data were collected, which included sexuality, age, marital status, income, employment status, occupation, psychiatric disorder comorbidities, family history of psychiatric disorders; health issues, namely hypertension, hyperlipidemia, hyperglycemia, personality type, cigarette or alcohol use, and body mass index.

Second, COVID-19 related data were collected, namely whether the participant (1) was a confirmed/suspected/asymptomatic case of COVID-19; (2) had acquaintances who were infected with or died from COVID-19; (3) had a history of visiting Wuhan, and if so, (4) the duration of stay in that region; and (5) worries regarding extensive re-outbreak of COVID-19.

Third, the respondents' mental health was evaluated *via* the (1) Impact of Event Scale-Revised (IES-R, for evaluation of vicarious traumatization relevant to COVID-19 pandemic), (2) Self-Rating Depression Scale (SDS, for evaluation of depression), (3) Self-Rating Anxiety Scale (SAS, for evaluation of anxiety), (4) Obsessive-Compulsive Inventory-Revised (OCI-R, for evaluation of obsessions and compulsions), (5) Pittsburgh Sleep Quality Index-Revised (PSQI-R, for evaluation of sleeping quality), (6) Social Support Rating Scale (SSRS, for evaluation of social support level), (7) Beck Suicide Ideation Scale (SSI, for evaluation of suicidal ideation and attempts), and (8) 12-Item Short-Form Health Survey (SF-12, for evaluation of quality of life). Supplementary Material 3 details the full information regarding psychometric evaluation and the purpose of each metric.

All respondents were informed of the purpose and processes of the study. Written consent was collected at the beginning of the online questionnaire, and participants voluntarily completed the survey. Oral approval of study conduct was received from the ethics committee of Renmin Hospital of Wuhan University.

### Statistical Analysis

SPSS 24.0 software (IBM Corp.; Armonk, NY) was used for all statistical analysis.

#### Descriptive Population

In our pilot survey a detection rate of 25% was found to be inpatients with pVT, according to the equation *N* = *k* × (1–*P*)/*P*, when *k* was valued as 100, *N* was equal to 300. Due to the restriction and control of beds in hospital, as well as a strict standard for inclusion and exclusion, 266 inpatients were deemed as eligible.

#### Correlation and Regression Analysis

We defined a binary dependent variable, namely non-pVT and pVT. First, univariate analyses were conducted to determine variable potentially related to pVT. A chi square test was used for comparison of differences between groups of categorical data. For continuous variables, normal distribution of values was first tested. Variables that conformed to the normal distribution were compared by Students' *t*-test; otherwise, a non-parametric test was used. Subsequently, variables identified as potentially related to pVT entered a multi-factorial regression model. Our statistical significance threshold was defined as a *p* < 0.05.

## Results

### Basic Information

Information relevant to population was recorded in Table 1, in which categorical variables were described in the form of *N* and percentage, numerical variables in the form of Mean ± SD. In total, 454 inpatients were invited to participate in the study, of whom 277 completed the questionnaire, for a completion proportion rate of 61.01%. In the later verification phase, one respondent was identified as having employed another person to answer the questionnaire, three persons gave the wrong answer to the decoy question, and six had previously been infected with COVID-19; finally, 266 eligible patients remained. 74.06% of the sample were female. The average age was 30.91 ± 16.58 years, ranging from 14 to 73 years old. Most participants had low monthly income (<3k). Among the sample, 62.41% were employed or at school, while the remaining 100 reported they were unemployed or had retired. Nearly half of the cohort was composed of students. Inpatients from the psychological department represented 65.79% of the sample, with the remainder from the psychiatric department. Approximately 70% reported a psychiatric comorbidity. Forty-three subjects reported a family history of psychiatric problems and 34 admitted a history of common chronic disease. Twelve participants stated they had acquaintances who were infected with or died from COVID-19. Nearly half of the sample were introverted in personality and most did not use alcohol or tobacco. History of visiting Wuhan was 2.8 ± 4.3 days on average, while the average duration of stay in Wuhan was 90.0 ± 131.9 days on average. Over half of the sample were worried on re-outbreak of the epidemic domestically. Notably, 2/3 or so were reported to have suicidal ideation (Table 1). Check out Table 1 for detailed information.

**Table 1:** Socio-demographic characteristics captured in our sample of study.

**Variables**	**Classification**	**Parameter**
Sexuality	Male	69 (25.94)
	Female	197 (74.06)
Marital status	No	157 (59.02)
	Yes	109 (40.98)
Income (RMB)	<3k	192 (72.18)
	3–5k	41 (15.41)
	>5k	33 (12.41)
Employment status	Employed	54 (20.30)
	At school	112 (42.11)
	Retired	26 (9.77)
	Unemployed	74 (27.82)
Occupation	Students	117 (43.98)
	Non-students	149 (56.02)
Department	Psychological department	175 (65.79)
	Psychiatric department	91 (34.21)
Comorbidities	No	79 (29.70)
	Yes	187 (70.30)
Family history	No	223 (83.83)
	Yes	43 (16.16)
Chronic diseases	No	232 (87.22)
	Yes	34 (12.78)
Acquaintances infected with or died from COVID-19	No	254 (95.49)
	Yes	12 (4.51)
Disposition	Introverted	120 (45.11)
	Impartial	88 (33.09)
	Extraverted	58 (21.80)
Cigarette or alcohol use	No	224 (84.21)
	Cigarettes	13 (4.89)
	Alcohol	13 (4.89)
	Both	16 (6.02)
Worries regarding re-outbreak	No	118 (44.36)
	Yes	148 (55.64)
Suicidal ideation	No	96 (36.09)
	Yes	170 (63.91)
Latency of VTS (months)	<1	132 (49.62)
	1–3	33 (12.41)
	3–6	22 (8.27)
	>6	80 (30.08)
Duration of VTS (months)	<1	137 (51.50)
	1–3	52 (19.55)
	>3	77 (28.95)
Age		30.91 (±16.58)
BMI		21.55 (±3.30)
Visit history		2.8 (±4.3)
Duration of stay(days)		90.0 (±131.9)
IES-R		23.57 (±16.45)
A		7.19 (±6.36)
I		7.41 (±5.87)
H		8.97 (±5.78)
SDS		62.36 (±15.16)
SAS		54.29 (±13.03)
OCI-R		21.02 (±15.41)
Washing		2.59 (±2.84)
Obsession		3.89 (±2.90)
Hoarding		3.32 (±2.83)
Ordering		4.24 (±3.19)
Checking		3.66 (±3.11)
Miscellaneous		3.30 (±2.97)
SSI (ideation)		8.61 (±3.30)
SSI (attempts)		29.42 (±23.29)
SF-12		66.71 (±21.41)
PSQI		11.02 (±3.55)
Satisfaction		2.69 (±0.96)
Disturbance		3.10 (±1.02)
Latency		2.91 (±1.15)
Duration		2.32 (±1.14)
SSRS		33.08 (±8.89)
Subjective		19.24 (±5.59)
Objective		7.36 (±3.19)
Availability		6.48 (±2.11)

### Characteristics of Suspected Vicarious Traumatization

**Figure 1:**
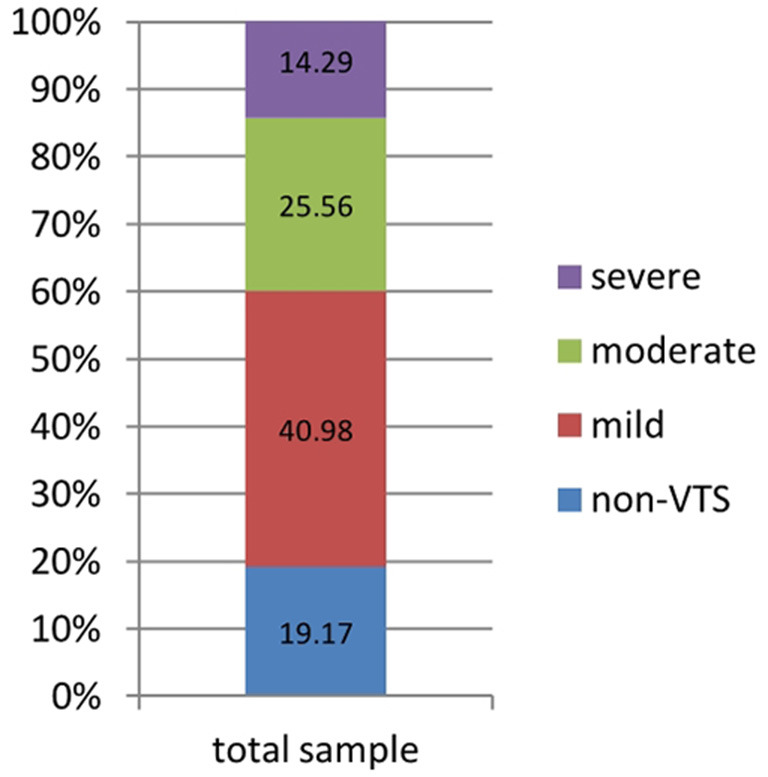
Distribution of different levels of VTS among inpatients.

Overall, 215 respondents scored up to 9 points, such that VTS was present in 80.83% of the sample, with 40.98% reporting mild symptoms, 25.56% moderate, and 14.29% severe (Table 2 and Figure 1). Among all subjects with VTS, 211 presented all three types of symptomatic dimensions, 13 presented two types, and only one presented single a single dimension of “H.”

**Table 2:** Distribution of symptoms among respondents with suspected vicarious traumatization.

**Dimension of VTS**	***N***	**Percentage, %**
A	0	0
I	0	0
H	1	0.47
A+I	1	0.47
A+H	2	0.93
I+H	10	4.65
A+I+H	201	93.48

*I, intrusion; H, hypervigilance; A, avoidance*.

Figure 1 gave an account of corresponding detection rate of different levels of VTS, in which section blue, red, green, purple represented non-VTS, mild VTS, moderate VTS and severe VTS, respectively.

### Risk Factors Associated With pVT

As indicated in the univariate analyses, pVT was more frequent in retired (50%) and unemployed persons (29.37%) than in employed persons (16.67%) and those in school (25.00%; *p* = 0.015). Higher frequency of pVT was present in inpatients with comorbidities than in those without comorbidities (*p* < 0.001). The proportion of cases of pVT was greater among inpatients with acquaintances infected with or had died from COVID-19 than among those without such acquaintances (*p* = 0.002). The proportion was higher among those who expressed worrying regarding re-outbreak of the epidemic vs. other persons (*p* < 0.001).

OCI-R scores of patients with pVT were higher than those of patients without pVT (*p* < 0.001), as were SSI (ideation) scores (*p* = 0.001), SSI (attempts) scores (*p* = 0.003), PSQI-R scores (*p* < 0.001), SAS scores (*p* < 0.001), and SDS scores (*p* < 0.001). SF-12 scores of patients with pVT were lower than those of other patients (*p* < 0.001), as were SSRS scores (*p* = 0.040). Inpatients with a VTS latency of 1-3 months had the highest prevalence of pVT (*p* < 0.001), and those with longer duration of VTS had a greater prevalence of pVT (*p* < 0.001; Table 3).

**Table 3:** Comparison of mental health status between respondents with or without pVT.

**Variables**		**Non-pVT, *N***	**pVT, *N***	***p***
Total		194	72	
Sex	Male	55	14	0.141
	Female	139	58	
Age		30.26 ± 15.48	32.68 ± 19.24	0.939
Marital status	Unmarried	117	40	0.484
	Married	77	32	
Income (RMB)	<3k	140	52	0.890
	3–5k	29	12	
	>5k	25	8	
Employment	Employed	45	9	0.015
	At school	84	28	
	Retired	13	13	
	Unemployed	52	22	
Occupation	Students	86	31	0.852
	Non-students	108	41	
Department	Psychological	132	43	0.204
	Psychiatric	62	29	
Medical insurance	Hubei	98	34	0.195
	Wuhan	50	26	
	Self-paying	46	12	
Comorbidities	No	131	16	<0.001
	Yes	63	56	
Family history	No	164	59	0.610
	Yes	30	13	
Chronic diseases	No	173	59	0.117
	Yes	21	13	
Acquaintances infected with or died from COVID-19	No	190	64	0.002
	Yes	4	8	
Personality	Introverted	89	31	0.815
	Impartial	62	26	
	Extraverted	43	15	
Cigarette or alcohol use	No	164	60	0.811
	Yes	30	12	
Worries regarding re-outbreak	No	104	14	<0.001
	Yes	90	58	
Suicidal ideation	No	74	22	0.252
	Yes	120	50	
Latency of VTS	<1	114	17	<0.001
	1-3	16	17	
	3-6	12	10	
	>6	52	28	
Duration of VTS	<1	120	17	<0.001
	1-3	33	19	
	>3	41	36	
Age		30.26 ± 15.48	32.68 ± 19.24	0.939
BMI		21.53 ± 3.32	21.59 ± 3.28	0.791
Visit history		2.52 ± 4.17	3.65 ± 4.74	0.085
Duration of stay (days)		79.5 ± 125.5	118.3 ± 144.9	0.092
OCI-R		17.12 ± 13.46	31.53 ± 15.53	<0.001
SSI (ideation)		8.14 ± 3.00	9.89 ± 3.75	0.001
SSI (attempt)		26.30 ± 20.93	37.83 ± 27.14	0.003
SF-12		70.93 ± 21.23	55.35 ± 17.48	<0.001
PSQI		10.52 ± 3.59	12.39 ± 3.09	<0.001
SSRS		33.68 ± 9.44	31.47 ± 6.98	<0.040
SAS		51.18 ± 11.52	62.67 ± 13.25	<0.001
SDS		58.81 ± 14.34	71.90 ± 13.11	<0.001

Variables significant in the univariate analysis were entered in the multi-factorial logistic regression (Forward entry). As shown in Table 4, pVT was independently associated with inpatients with acquaintances who had been infected with or died from COVID-19, worries of re-outbreak of the epidemic, higher OCI-R, lower SF-12 scores, and longer latency of VTS were independently correlated with pVT.

**Table 4:** Multi-factorial logistic regression of variables relevant to pVT.

	**Variable**	**B**	**SD**	**Wald**	***P***	**Exp (B)**	**95% CI**
Step1	OCI-R	0.063	0.010	37.302	<0.001^*^	1.065	1.044–1.087
	Common	−2.481	0.302	67.417	<0.001^*^	0.084	
Step2	Worries regarding re-outbreak	1.558	0.365	18.184	<0.001^*^	4.748	2.320–9.715
	OCI-R	0.063	0.011	33.576	<0.001^*^	1.065	1.043–1.088
	Common	−5.084	0.748	46.253	<0.001^*^	0.006	
Step3	Worries regarding re-outbreak	1.533	0.373	16.881	<0.001^*^	4.634	2.230–9.629
	OCI-R	0.056	0.011	23.817	<0.001^*^	1.057	1.034–1.081
	SF-12	−0.032	0.009	11.891	0.001^*^	0.968	0.951–0.986
	Common	−2.850	0.942	9.150	0.002^*^	0.058	
Step4	Infected or deceased acquaintances	2.967	0.933	10.108	0.001^*^	19.427	3.120–120.976
	Worries regarding re-outbreak	1.666	0.395	17.772	<0.001^*^	5.293	2.439–11.485
	OCI-R	0.058	0.012	23.388	<0.001^*^	1.059	1.035–1.084
	SF12	−0.034	0.010	13.048	<0.001^*^	0.966	0.948–0.984
	Common	−6.090	1.442	17.829	<0.001^*^	0.002	
Step5	Infected or deceased acquaintances	3.109	1.023	9.238	0.002^*^	22.388	3.016–166.182
	Worries on re-outbreak	1.515	0.403	14.167	<0.001^*^	4.550	2.067–10.014
	OCI-R	0.051	0.012	16.627	<0.001^*^	1.052	1.027–1.078
	SF-12	−0.035	0.010	10.205	0.001^*^	0.966	0.947–0.985
	Latency of VTS			9.276	0.026^*^		
	Latency (1)	1.410	0.530	7.071	0.008^*^	4.095	1.449–11.573
	Latency (2)	1.239	0.580	4.561	0.033^*^	3.450	1.107–10.752
	Latency (3)	0.818	0.416	3.869	0.049^*^	2.266	1.003–5.121
	Common	−6.423	1.519	17.876	0.000^*^	0.002	

* *p < 0.05*.

## Discussion

Far from only a disease, COVID-19 has become a social event that has negatively affected people both directly or indirectly. To the best of our knowledge, this is the first study to focus on vicarious traumatization of psychiatric inpatients during the remission phase of the pandemic.

Although PTSD and VT were reported shortly after the outbreak of COVID-19 (15, 16), such conditions can persist. A Japanese researcher reported that nearly 7% of relief workers had probable PTSD 1 year after the Great East Japan Earthquake (17). In our study, fully 80% of the inpatients were experiencing VTS due to the pandemic 6 months after the lockdown was lifted, among whom 27.07% were considered to exhibit pVT. There may be two main reasons for such a high detection rate. The first relates to the chronic nature of stress disorders. Respondents may experience stress at the beginning of a precipitating event, but the level or magnitude may be too minor to be sensed or detected. However, with time, such stress might build until it finally exerts a dominant effect. Repeated and continuous exposure to stress is not unusual since the pandemic situation is continually variable. Second, psychiatric patients themselves are often susceptible to stress (18); up to 98% of patients with severe mental diseases may be victimized by the attendant trauma. This emphasizes that attention should be paid to VT among psychiatric inpatients in clinical settings.

In our study, two risk factors were associated with pVT, namely having acquaintances who had been infected with or died from COVID-19 and worries regarding re-outbreak of the pandemic. These represent concrete sources of stress from the past and stress toward the future, although both were indirect because the respondents had never personally contracted COVID-19. People might receive news from different channels, such as Internet, TV shows, and newspapers, which might even present unconfirmed rumors. Future studies should address whether there are any relationships between how information is obtained and occurrence of VT.

Our results showed that respondents with 1–3 months of symptomatic latency were more likely to exhibit pVT compared with latency of <1 month, but longer latency did not indicate a greater level of risk. This may be attributed to the progressive development of a psychiatric disorder like VT or PTSD; likely the symptoms of those with longer latency were newly emerging and the documented severity of VT was below threshold, given that a relatively strict criterion for determination of pVT was applied according to a previous study (19). Correlations between latency and severity of different phenotypes of symptoms would be worth further investigation.

Recently a community study in Italy found the mean SF-12 score in people with PTSD was lower than that in people without PTSD, and some disorders were often comorbid with PTSD, such as major depressive disorder, panic disorder, and bipolar spectrum disorders (20). Our results revealed similar tendencies, whereby symptoms such as depression, anxiety, and obsession or compulsion were more severe in patients with pVT rather than non-pVT, as were levels of sleep quality and social support. However, in the multivariate regression, only SF-12 and OCI-R scores were independently associated with pVT. This finding is novel; we suggest two explanations. First, the timing, methods, selected target, as well as scales differed between studies. Second, interactions between independent variables could have exerted a synthetic effect, as unknown variables with potential influence were not included in the current study. Simultaneously, as reported by Franklin in a survey of veterans (21), similarities of partial symptoms between OCD and PTSD make it difficult to determine how these symptoms are connected; intermediate factors may exist, which calls for more research in the future.

Interestingly, among patients with VTS, the type of symptoms were consistent; over 93% of patients with VTS possessed all three phenotypes. This phenomenon strongly indicates that intrusion, avoidance, and hypervigilance are not independent of each other, but rather belong to a sequential process of VT or PTSD, or they could intimately interact with each other forming a vicious cycle. In contrast, Co-occurrence of symptoms also makes it easier to identify the disease, if appropriate attention is given.

Notably, Auxemery also highlighted that ambiguity and complexity exist in distinguishing descriptions of post-traumatic psychiatric disorders. Example descriptions are post traumatic stress disorder, secondary traumatization, compassion fatigue, and vicarious traumatization, as used in the current study. In this respect, the specification that stress was pandemic-related rather than from other life events was highlighted in the IES-R. Further, inpatients with a history of COVID-19 virus infection were excluded to guarantee the quality of the sample to the maximum extent.

### Limitations and Prospects

First, policies restricting the capacity of hospital beds limited our ability to recruit additional patients; we are planning to collect more data in the future.

Second, the study's cross-sectional design does not permit the inference of causal relationships among variables. However, the data provide a foundation for subsequent work and a follow-up study in 1 or 2 years' time may permit a longitudinal comparison.

Third, a deeper analysis of correlations among different symptoms of sub-dimensions was not undertaken due to the limited sample size; we hope to address this problem in the future.

## Conclusions

Even in the phase of remission, COVID-19 can have a profound influence on the mental state of psychiatric inpatients. It is therefore important to conduct screening in wards for patients at risk, to enable timely intervention in cases of vicarious traumatization or other psychiatric problems.

## Data Availability Statement

The raw data supporting the conclusions of this article will be made available by the authors, without undue reservation.

## Ethics Statement

The studies involving human participants were reviewed and approved by the ethics committee of Renmin Hospital of Wuhan University. Written informed consent to participate in this study was provided by the participants' legal guardian/next of kin.

## Author Contributions

YZ, LX, and YH: design and conception. GW, HW, and LX: modification and collation. YZ: paper writing and statistical analysis. QW and YX: liaison and mobilization on publics. YZ and YX: collection and distribution of questionnaire. All authors contributed to the article and approved the submitted version.

## Conflict of Interest

The authors declare that the research was conducted in the absence of any commercial or financial relationships that could be construed as a potential conflict of interest.

## Publisher's Note

All claims expressed in this article are solely those of the authors and do not necessarily represent those of their affiliated organizations, or those of the publisher, the editors and the reviewers. Any product that may be evaluated in this article, or claim that may be made by its manufacturer, is not guaranteed or endorsed by the publisher.

## Abbreviations

VT: Vicarious Traumatization
VTS: vicarious traumatic symptoms
pVT: possible vicarious traumatization
SAS: Self-Rating Anxiety Scale
SDS: self-rating depression scale
SSRS: social support rating scale
IES-R: Impact of Event Scale-Revised
OCI-R: Obsessive-Compulsive Inventory-Revised
PSQI-R: Pittsburgh Sleep Quality Index-Revised
SSI: Beck Suicide Ideation Scale
SF-12: 12-Item Short-Form Health Survey
PTSS: post-traumatic stress symptoms
PTSD: post-traumatic stress disorders
SARS: severe acute respiratory syndrome
I: intrusion
H: hypervigilance
A: avoidance.
