# Supplementary material for: Possible Vicarious Traumatization Among Psychiatric Inpatients During the Remission Phase of the COVID-19: A Single-Center Cross-Sectional Study
Source: Front Psychiatry. 2021 Aug 24;12:677082. doi: 10.3389/fpsyt.2021.677082 (PMC8421644; doi:10.3389/fpsyt.2021.677082)
Supplement: Supplementary file 1 [file Data_Sheet_1.pdf]

常态化阶段新冠肺炎公共卫生事件影响及心理健康问卷

尊敬的病友，您好，19 年底一场突如其来的新冠肺炎给全人类带来了重大的灾难，不仅给人们身心造成危害，也为社会、经济发展蒙上阴影。虽然我们及时遏制住了病毒的传播，但其影响是深远的，为了更好的落实国家全病程防控策略以及疫后心理重建方针，武汉大学人民医院精神卫生中心免费为广大患者进行健康测试，以了解现阶段新冠肺炎事件对于重灾区武汉的住院患者产生心理困扰的强度和性质，以期及时对特定人群予以有目的、有针对、有条件的个体化治疗，维护个人和社会稳定。该问卷共 144 题，约花费 15-20 分钟，所有信息将严格保密，仅用于本次科学研究，请阅读指导语，放心填写，提交后生效。

——武汉大学人民医院精神卫生中心，湖北省神经精神病研究所

一、基本信息

本部分为 1-19 题，为年龄、性别、既往病史等一般资料。

1.性别 [单选题] *

○男

○女

2.年龄(岁) [填空题] *

____

3.婚姻状况 [单选题] *

○未婚

○已婚

4.经济水平(月收入) [单选题] *

○不足 1 千

○1-3 千

○3-5 千

○5-1 万

○1 万以上

5.工作状况 [单选题] *

○在业

○在学

○离退休

○无业/失业

6.职业类型 [单选题] *

○教师

○学生

○农民

○工人

○医护人员

○公务员

○其他事业单位人员

○其他企业单位人员

○个体

○其他

7. 7+12= [单选题] *

○13

○8

○19

○9

8.是否感染过新冠 [单选题] *

○否

○无症状感染

○疑似病例

○确诊病例

9.是否确诊过其他心理疾患 [单选题] *

○无

○精神分裂症

○抑郁症

○躁狂症

○双向障碍

○焦虑症

○强迫症

○物质依赖

○急、慢性应激障碍

○其他

10.是否有心理疾患家族史 [单选题] *

○无

○精神分裂症

○抑郁症

○躁狂症

○双向障碍

○焦虑症

○强迫症

○物质依赖

○急、慢性应激障碍

○其他

11.是否有常见慢性疾病史(如高血压、冠心病、糖尿病等) [单选题] *

○否

○是

12.是否有亲朋好友感染过新冠肺炎 [单选题] *

○否

○是

13.是否有亲朋好友因感染新冠肺炎而死亡 [单选题] *

○否

○是

14.平素性格 [单选题] *

○偏内向

○中等

○偏外向

15.烟酒史 [单选题] *

○不抽烟不喝酒

○抽烟但不喝酒

○喝酒但不抽烟

○抽烟又喝酒

16.身高：_________厘米　体重：________千克 [填空题] *

17.疫情爆发以来往返武汉次数（填数字即可，如 1 代表 1 次，一直没离开武汉写 0） [填空题] *

____

18.疫情爆发以来在武汉总逗留_________ 个月 _________天 [填空题] *

19.是否担心国内新冠肺炎卷土重来？ [单选题] *

○否

○是

二、心理健康部分

20-99 题，该部分主要从创伤应激障碍、抑郁、焦虑、强迫这几个方面来衡量您目前的心理状况。

新冠所引起的创伤体验（请根据此次新冠肺炎国内爆发以来，您的主观感受进行描述）

20.任何与新冠肺炎有关的事物(如看到新闻报道，看到口罩消毒液、去医院看病)都会引发我疫情爆发时的感受，好像疫情就在我眼前一样 [单选题] *

○无

○很少

○有时

○经常

○总是

21.因为疫情，我很难安稳的一觉睡到天亮 [单选题] *

○无

○很少

○有时

○经常

○总是

22.一些平时与疾病不相关、毫无意义的东西也会让我想起新冠肺炎 [单选题] *

○无

○很少

○有时

○经常

○总是

23.因为疫情我感觉易受刺激、易发怒 [单选题] *

○无

○很少

○有时

○经常

○总是

24.每当想起新冠肺炎或遇到一些使我想起它的事情，我会尽量竭力避免、逃避防止自己心烦意乱 [单选题] *

○无

○很少

○有时

○经常

○总是

25.即使我不愿去想起新冠肺炎，也会想起它 [单选题] *

○无

○很少

○有时

○经常

○总是

26.我感觉，新冠肺炎好像不是真的，或者从未发生过 [单选题] *

○无

○很少

○有时

○经常

○总是

27.我设法远离一切能使我记起新冠肺炎的事物 [单选题] *

○无

○很少

○有时

○经常

○总是

28.有关新冠肺炎的一些画面如医生救人场景、病人家属生离死别等场景反复再我的脑海中突然出现 [单选题] *

○无

○很少

○有时

○经常

○总是

29.我感觉疫情搞得自己神经过敏，易被惊吓，做什么都很小心谨慎，畏手畏脚 [单选题] *

○无

○很少

○有时

○经常

○总是

30.我努力克制不去想新冠疫情 [单选题] *

○无

○很少

○有时

○经常

○总是

31.我感觉到自己对新冠肺炎疫情有诸多感受，但我没有办法去处理这些问题 [单选题] *

○无

○很少

○有时

○经常

○总是

32.我对疫情的感觉有些麻木了 [单选题] *

○无

○很少

○有时

○经常

○总是

33.我发现自己的行为和感觉，好像又回到了疫情刚刚爆发的那个时候一样，熟悉又陌生 [单选题] *

○无

○很少

○有时

○经常

○总是

34.疫情让我难以入睡 [单选题] *

○无

○很少

○有时

○经常

○总是

35.新冠肺炎常使我发生强烈的情感波动 [单选题] *

○无

○很少

○有时

○经常

○总是

36.我想要忘掉这次新冠疫情 [单选题] *

○无

○很少

○有时

○经常

○总是

37.我感觉疫情使得自己难以集中注意力 [单选题] *

○无

○很少

○有时

○经常

○总是

38.那些使我想起新冠疫情的事物会给我带来身体不适，如：出汗、心慌胸闷、头晕眼花、尿频尿急、便秘腹泻等 [单选题] *

○无

○很少

○有时

○经常

○总是

39.我曾做梦梦到过自己或亲人得了新冠肺炎或死去 [单选题] *

○无

○很少

○有时

○经常

○总是

40.我感觉自己很警觉、很戒备，对什么人都不放心 [单选题] *

○无

○很少

○有时

○经常

○总是

41.我尽量不去再提新冠肺炎 [单选题] *

○无

○很少

○有时

○经常

○总是

您首次发生以上症状（20-41 题）距离国内新冠爆发（19 年 12 月底）多久？ [单选题] *

○很短

○不到 1 个月

○1-3 个月

○3-6 个月

○超过半年

您出现上述症状总共持续时间多长 [单选题] *

○很短

○不到 1 个月

○1-3 个月

○超过 3 个月

抑郁

42.我觉得闷闷不乐，情绪低沉。 [单选题] *

○很少

○少部分时间

○相当多的时间

○绝大多数时间

43.我觉得一天之中早晨最好。 [单选题] *

○很少

○少部分时间

○相当多的时间

○绝大多数时间

44.我一阵阵哭出来或觉得想哭。 [单选题] *

○很少

○小部分时间

○相当多的时间

○绝大部分时间

45.我晚上睡眠不好。 [单选题] *

○很少

○小部分时间

○相当多的时间

○绝大部分时间

46.我吃得跟平常一样多。 [单选题] *

○很少

○小部分时间

○相当多的时间

○绝大部分时间

47.我与异性密切接触时和以往一样感到愉快。 [单选题] *

○很少

○小部分时间

○相当多的时间

○绝大部分时间

48.我发觉我的体重在下降。 [单选题] *

○很少

○小部分时间

○相当多的时间

○绝大部分时间

49.我有便秘的苦恼。 [单选题] *

○很少

○小部分时间

○相当多的时间

○绝大部分时间

50.我心跳比平时快。 [单选题] *

○很少

○小部分时间

○相当多的时间

○绝大部分时间

51.我无缘无故的感到疲乏。 [单选题] *

○很少

○小部分时间

○相当多的时间

○绝大部分时间

52.我的头脑跟平常一样清楚。 [单选题] *

○很少

○小部分时间

○相当多的时间

○绝大部分时间

53.我觉得自己应对以前经常做的事情并没有困难。 [单选题] *

○很少

○小部分时间

○相当多的时间

○绝大部分时间

54.我觉得不安而平静不下来。 [单选题] *

○很少

○小部分时间

○相当多的时间

○绝大部分时间

55.我对将来抱有希望。 [单选题] *

○很少

○小部分时间

○相当多的时间

○绝大部分时间

56.我比平常容易生气激动。 [单选题] *

○很少

○小部分时间

○相当多的时间

○绝大部分时间

57.我觉得作出决定是容易的。 [单选题] *

○很少

○小部分时间

○相当多的时间

○绝大部分时间

58.我觉得自己是个有用的人，有人需要我。 [单选题] *

○很少

○小部分时间

○相当多的时间

○绝大部分时间

59.我的生活过的很有意思。 [单选题] *

○很少

○小部分时间

○相当多的时间

○绝大部分时间

60.我认为如果我死了别人会生活得好些。 [单选题] *

○很少

○小部分时间

○相当多的时间

○绝大部分时间

61.平常感兴趣的事我仍然照样感兴趣。 [单选题] *

○很少

○小部分时间

○相当多的时间

○绝大部分时间

焦虑

62．我觉得比平时容易紧张和着急 [单选题] *

○很少

○小部分时间

○相当多的时间

○绝大部分时间

63. 我无缘无故地感到害怕 [单选题] *

○很少

○小部分时间

○相当多的时间

○绝大部分时间

64．我容易心里烦乱或觉得惊恐 [单选题] *

○很少

○小部分时间

○相当多的时间

○绝大部分时间

65．我觉得我可能将要发疯 [单选题] *

○很少

○小部分时间

○相当多的时间

○绝大部分时间

66．我觉得一切都很好，也不会发生什么不幸 [单选题] *

○很少

○小部分时间

○相当多的时间

○绝大部分时间

67．我手脚发抖打颤 [单选题] *

○很少

○小部分时间

○相当多的时间

○绝大部分时间

68．我因为头痛、颈痛和背痛而苦恼 [单选题] *

○很少

○小部分时间

○相当多的时间

○绝大部分时间

69．我感觉容易衰弱和疲乏 [单选题] *

○很少

○小部分时间

○相当多的时间

○绝大部分时间

70．我觉得心平气和，并且容易安静坐着 [单选题] *

○很少

○小部分时间

○相当多的时间

○绝大部分时间

71．我觉得心跳得快 [单选题] *

○很少

○小部分时间

○相当多的时间

○绝大部分时间

72．我因为一阵阵头晕而苦恼 [单选题] *

○很少

○小部分时间

○相当多的时间

○绝大部分时间

73．我有过晕倒发作，或觉得要晕倒似的 [单选题] *

○很少

○小部分时间

○相当多的时间

○绝大部分时间

74．我呼气吸气都感到很容易 [单选题] *

○很少

○小部分时间

○相当多的时间

○绝大部分时间

75．我手脚麻木和刺痛 [单选题] *

○很少

○小部分时间

○相当多的时间

○绝大部分时间

76．我因胃痛和消化不良而苦恼 [单选题] *

○很少

○小部分时间

○相当多的时间

○绝大部分时间

77．我常常要小便 [单选题] *

○很少

○小部分时间

○相当多的时间

○绝大部分时间

78．我的手常常是干燥温暖的 [单选题] *

○很少

○小部分时间

○相当多的时间

○绝大部分时间

79．我脸红发热 [单选题] *

○很少

○小部分时间

○相当多的时间

○绝大部分时间

80．我容易入睡并且一夜睡得很好 [单选题] *

○很少

○小部分时间

○相当多的时间

○绝大部分时间

81．我做恶梦 [单选题] *

○很少

○小部分时间

○相当多的时间

○绝大部分时间

强迫

82.我囤积了很多东西(如口罩、消毒水、防护面罩等)，即使知道可能比较过度，但仍就是忍不住。 [单选题] *

○一点都没有

○一点点

○中等

○很多

○极多

83.不管有没有需要我都要重复检查(比如反复检查门锁好没有、东西拿好没有、纽扣系错没有等)，生怕遗漏什么东西。 [单选题] *

○一点都没有

○一点点

○中等

○很多

○极多

84.如果事情没有安排妥当或计划周全我会心烦意乱，浑身不舒服。 [单选题] *

○一点都没有

○一点点

○中等

○很多

○极多

85.我做事情的时候会强迫自己去数数，经常数到某个数又怀疑自己数错了，便重头来过。 [单选题] *

○一点都没有

○一点点

○中等

○很多

○极多

86.如果我知道一样东西被陌生人或谁碰过我就很难接受让自己再去碰 [单选题] *

○一点都没有

○一点点

○中等

○很多

○极多

87.我很难控制自己的思想 [单选题] *

○一点都没有

○一点点

○中等

○很多

○极多

88.我会收集自己并不需要的东西 [单选题] *

○一点都没有

○一点点

○中等

○很多

○极多

89.我反复检查门、窗、抽屉等。 [单选题] *

○一点都没有

○一点点

○中等

○很多

○极多

90.如果别人改变了我计划好的事情或改变我放东西的位置我会觉得心烦意乱 [单选题] *

○一点都没有

○一点点

○中等

○很多

○极多

91.我觉得自己必须重复读、写、记住某些数字。 [单选题] *

○一点都没有

○一点点

○中等

○很多

○极多

92.有时候我仅仅因为自己觉得身上脏就反复去清洗（即使旁人觉得还好，因此不被人理解）。 [单选题] *

○一点都没有

○一点点

○中等

○很多

○极多

93.当有和我意愿相反的想法时我会觉得心烦意乱。 [单选题] *

○一点都没有

○一点点

○中等

○很多

○极多

94.我不丢弃物品，因为我怕它们以后还有用。 [单选题] *

○一点都没有

○一点点

○中等

○很多

○极多

95.在关了门、窗、煤气、电灯开关之后，我还是认为没有关好，要反复检查。 [单选题] *

○一点都没有

○一点点

○中等

○很多

○极多

96.我必须按特定的规则或次序来摆放东西。 [单选题] *

○一点都没有

○一点点

○中等

○很多

○极多

97.我觉得数字有好坏之分，相信存在幸运或晦气数字。 [单选题] *

○一点都没有

○一点点

○中等

○很多

○极多

98.我洗手的时间和频率都比实际需要的多得多，但无法控制。 [单选题] *

○一点都没有

○一点点

○中等

○很多

○极多

99.我经常有些污秽、有关性的想法和念头，而且很难摆脱。 [单选题] *

○一点都没有

○一点点

○中等

○很多

○极多

第三部分

100-143 本部分主要评估您睡眠、社会支持、生活质量、自杀意念等状况。

睡眠

100.近 1 个月来，总的来说您觉得自己的睡眠质量怎么样？ [单选题] *

○很好

○较好

○较差

○很差

101.近 1 个月来，您夜间易醒、早醒的次数 [单选题] *

○无

○每周不足 1 次

○每周 1-2 次

○每周 3 次以上

102.近 1 个月来，您是否有入睡困难的情况(30 分钟不能入睡) [单选题] *

○无

○每周不足 1 次

○每周 1-2 次

○每周 3 次以上

103.近 1 个月来，您每天睡眠的平均时间多少 [单选题] *

○7 小时以上

○6-7 小时

○5-6 小时

○不足 5 小时

社会支持

104.您有多少关系密切，可以得到支持和帮助的朋友？ [单选题] *

○1 个也没有

○1-2 个

○3-5 个

○6 个或 6 个以上

105.近一年来您：(只选一项) [单选题] *

○远离家人，且独居一室

○住处经常变动，多数时间和陌生人住在一起

○和同学、同事或朋友住在一起

○和家人住在一起

106.您与邻居：(只选一项) [单选题] *

○相互之间从不关心，只是点头之交

○遇到困难可能稍微关心

○有些邻居都很关心您

○大多数邻居都很关心您

107.您与同事：(只选一项) [单选题] *

○相互之间从不关心，只是点头之交

○遇到困难可能稍微关心

○有些同事很关心您

○大多数同事都很关心您

108.从家庭成员得到的支持和照顾（在无、极少、一般、全力支持四个选项中，选择合适选项）[矩阵单选题] *

	无	极少	一般	全力支持
夫妻（恋人）	○	○	○	○
父母	○	○	○	○
儿女	○	○	○	○
兄弟姐妹	○	○	○	○
其他成员（如嫂子）	○	○	○	○

109.过去，在您遇到急难情况时，曾经得到的经济支持和解决实际问题的帮助的来源有： [多选题] *

□无任何来源

□配偶

□其他家人

□亲戚

□朋友

□同事

□工作单位

□党团工会等官方或半官方组织

□宗教、社会团体等非官方组织

□其它

110.过去，在您遇到急难情况时，曾经得到的安慰和关心的来源有：[多选题] *

□无任何来源

□配偶

□其他家人

□亲戚

□朋友

□同事

□工作单位

□党团工会等官方或半官方组织

□宗教、社会团体等非官方组织

□其它

111.您遇到烦恼时的倾诉方式：(只选一项) [单选题] *

○从不向任何人诉述.

○只向关系极为密切的 1-2 个人诉述。

○如果朋友主动询问您会说出来。

○主动诉述自己的烦恼，以获得支持和理解。

112.您遇到烦恼时的求助方式：(只选一项) [单选题] *

○只靠自己，不接受别人帮助。

○很少请求别人帮助。

○有时请求别人帮助。

○有困难时经常向家人、亲友、组织求援。

113.对于团体(如党团组织、宗教组织、工会、学生会等)组织活动，您：(只选一项) [单选题] *

○从不参加

○偶尔参加

○经常参加

○主动参加并积极活动。

自杀意念

114. 您希望活下去的程度如何？ * [单选题] *

○中等到强烈

○弱

○没有活着的欲望

115. 您希望死去的程度如何？ * [单选题] *

○没有死去的愿望

○弱

○中等到强烈

116. 您要活下去的理由胜过您要死去的理由吗？ * [单选题] *

○要活下去胜过要死去

○二者相当

○要死去胜过要活下来

117. 您主动尝试自杀的愿望程度如何？ * [单选题] *

○没有

○弱

○中等到强烈

118. 您希望外力结束自己生命，即有“被动自杀愿望”的程度如何？(如，希望一直睡下去不再醒来、意外地死去等) * [单选题] *

○没有

○弱

○中等到强烈

119. 您的这种自杀想法持续存在多长时间？ * [单选题] *

○短暂，一闪即逝

○较长时间

○持续或几乎是持续的

○近一周无自杀想法

120. 您的这种自杀想法持续存在多长时间？ * [单选题] *

○极少、偶尔

○有时

○经常或持续

○近一周无自杀想法

121. 您对自杀持什么态度？ * [单选题] *

○排斥

○矛盾或无所谓

○接受

122. 您觉得自己控制自杀想法、不把它变成行动的能力如何？ * [单选题] *

○能控制

○不知能否控制

○不能控制

123. 如果出现自杀想法，某些顾虑(如顾及家人、死亡不可逆转等)在多大程度上能阻止您自杀？ * [单选题] *

○能阻止自杀

○能减少自杀的风险

○无顾虑或无影响

124. 当您想自杀时，主要是为了什么？ * [单选题] *

○控制形势、寻求关注、报复

○逃避、减轻痛苦、解决问题

○前两种情况均有

○近一周无自杀想法

125. 您想过结束自己生命的方法了吗？ * [单选题] *

○没想过

○想过，但没制订出具体细节

○制订出具体细节或计划得很周详

126. 您把自杀想法落实的条件或机会如何？ * [单选题] *

○没有现成的方法、没有机会

○需要时间或精力准备自杀工具

○有现成的方法和机会或预计将来有方法和机会

○近一周无自杀想法

127. 您相信自己有能力并且有勇气去自杀吗？ * [单选题] *

○没有勇气、太软弱、害怕、没有能力

○不确信自己有无能力、勇气

○确信自己有能力、有勇气

128. 您预计某一时间您确实会尝试自杀吗？ * [单选题] *

○不会

○不确定

○会

129. 为了自杀，您的准备行动完成得怎样？ * [单选题] *

○没有准备

○部分完成(如，开始收集药片)

○全部完成(如，有药片、刀片、有子弹的*

130. 您已着手写自杀遗言了吗？ * [单选题] *

○没有考虑

○仅仅考虑、开始但未写完

○写完

131. 您是否因为预计要结束自己的生命而抓紧处理一些事情？如买保险或准备遗嘱。 * [单选题] *

○没有

○考虑过或做了一些安排

○有肯定的计划或安排完毕

132. 您是否让人知道自己的自杀想法？ * [单选题] *

○坦率主动说出想法

○不主动说出

○试图欺骗、隐瞒

○近一周无自杀想法

生活质量

133.总的来说，您认为您现在的健康状况是 [单选题] *

○非常好

○很好

○好

○一般

○差

134.在过去的四周，您是否因为情感方面的原因而不能做事或心不在焉？ [单选题] *

○有

○没有

135.以您目前的健康装看看，您在进行中等强度的活动时，有没有受到限制？例如搬桌子，打扫或清洁地板，打保龄球或打太极拳？ [单选题] *

○有很大限制

○有一点限制

○没有任何限制

136.以您目前的健康状况，是否影响您步行上楼？ [单选题] *

○有很大限制

○有一点限制

○没有任何限制

137.在过去四个星期，您会否因为身体健康原因，在日常生活或工作中感到力不从心？ [单选题] *

○会

○不会

138.在过去的四个星期的工作或日常生活中，您会否因为身体健康原因而令您的工作或活动受到限制？ [单选题] *

○会

○不会

139.在过去的四个星期，您会否因为情绪方面的原因(比如感到焦虑或沮丧)而令您的工作或活动受到限制？ [单选题] *

○会

○否

140.在过去的四个星期，您身体上的疼痛对您的日常工作(包括上班和家务)有多大影响？ [单选题] *

○完全没有影响

○有很少影响

○有一些影响

○有较大影响

○有非常大影响

141.在过去的四个星期里，您有多少时间感到心平气和？ [单选题] *

○常常

○大部分时间

○很多时间

○一般

○只有很少时间

○从来没有

142.在过去的四个星期里，您有多少时间感到精力充足？ [单选题] *

○常常

○大部分时间

○很多时间

○一般

○偶尔有

○从来没有

143.在过去的四个星期里，您有多少时间感到心情不好，闷闷不乐或沮丧？ [单选题] *

○常常

○大部分时间

○很多时间

○一般

○偶尔有

○从来没有

144.在过去的四个星期，有多少时间由于您身体健康或情绪问题而妨碍了您的社交活动(如探亲或访友)？ [单选题] *

○常常

○大部分时间

○很多时间

○一般

○偶尔有

○从来没有

URL：https://www.wjx.cn/?source=baidu&plan=%E9%97%AE%E5%8D%B7%E6%98%9F%EF%BC%88%E6%AD%A3%E5%B8%B8%EF%BC%89PC&keyword2=%E9%97%AE%E5%8D%B7%E6%98%9F%E5%93%81%E4%B8%93%E6%A0%87%E9%A2%98
