# Supplementary material for: Possible Vicarious Traumatization Among Psychiatric Inpatients During the Remission Phase of the COVID-19: A Single-Center Cross-Sectional Study
Source: Front Psychiatry. 2021 Aug 24;12:677082. doi: 10.3389/fpsyt.2021.677082 (PMC8421644; doi:10.3389/fpsyt.2021.677082)

WeChat and Wenjuanxin

One of the most popular apps for social interaction in China, iOS version 7.0.17-8.0.2 were adopted. Specifically, inpatients were informed of the purpose of the study before they were included into the trial. Afterwards, they would scan a uniform QR code to finish a questionnaire as depicted in supplementary material 1 via Wechat app. Instructions would be offered when necessary and patients could leave at any time if they felt reluctant. The whole process was voluntary. The QR code is as followed:

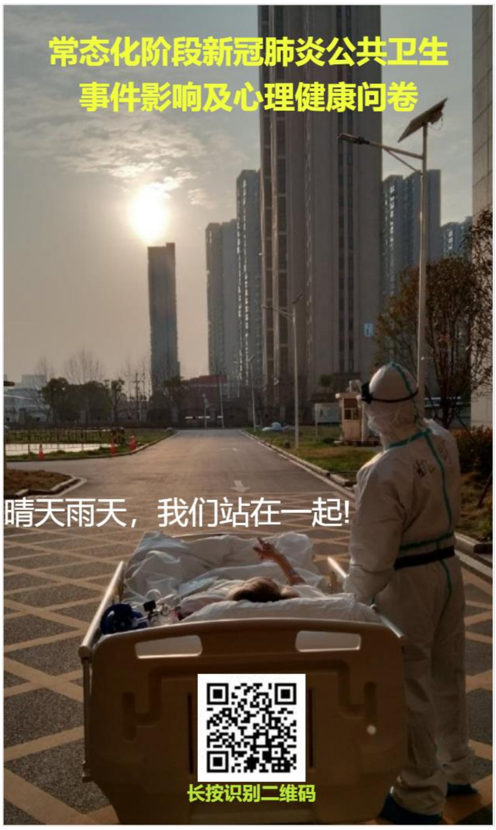

Supplement: Supplementary file 2 [file Data_Sheet_2.pdf]
