# Supplementary material for: Possible Vicarious Traumatization Among Psychiatric Inpatients During the Remission Phase of the COVID-19: A Single-Center Cross-Sectional Study
Source: Front Psychiatry. 2021 Aug 24;12:677082. doi: 10.3389/fpsyt.2021.677082 (PMC8421644; doi:10.3389/fpsyt.2021.677082)
Supplement: Supplementary file 3 [file Data_Sheet_3.pdf]

IES-R is a widely used scale to evaluate the level of post traumatic symptoms after experiencing unprecedented stress[1]. Both its validity and reliability have been confirmed in many studies[1]. As a classic Likert scale, it has 22 items, each item were counted from "0" to "4", representing an increasing frequency from "no" to " always ". 8 items belong to dimension of avoidance(A), another 8 items belong to dimension of intrusion (I) and the rest items belong to hypervigilance (H). A total score were added up by I,A ,H, ranging from 0 to 88. Generally, a score of 0 to 8 were considered as sub-clinical PTSS, 9 to 25 as mild, 26 to 43 as moderate and 44 to 88 as severe. According to a previous research, those scored up to 35 might be more like to be diagnosed with PTSD in the future[2]. Hence in our study, a cutoff point of 35 in IES-R were used to classify the sample into 2 groups, namely non-pVT(＜35) and pVT(⩾35).

SAS and SDS, both of which designed by W.K. Zung in 1965, are often used for the screening of anxious and depressed symptoms worldwide. The higher scores, the severer depression or anxiety[3].

OCI-R, a simplified version of OCI, was developed in 2002 by Foa[4]. 18 items were evenly classified into 6 dimensions as washing, obsession, hoarding, ordering, checking, miscellaneous. Each item has 5 options counted from "0" to "4", equivalent to different level of frequency from "not at all" to "always". Each dimension scores from 0-12 and total scores range from 0-78, the higher scores, the severer your obsessive compulsive symptoms. The Chinese version has been well-established with verification[5].

A modified version of PSQI were employed for assessment of sleep quality[6]. This scale contains 4 items representing 4 dimensions respectively. Item 1, used for reflection of subjective satisfaction of sleep quality, options from "very good" to "very bad" counted as 0-3, Item 2 for sleep disturbance, frequency from "no" to "over 3 times a week" counted as 0-3, item 3 for sleep latency, frequency from "no" to "over 3 times a week" counted as 0-3, and item 4 for sleep duration, sleeping hours from "above 7 hours" to "less than 5 hours" counted as 0-3.The total score would range from 0-21, a higher score indicates a poorer sleep quality.

The SSRS developed by Xiao was a practical inventory to evaluate level of social support, especially in Chinese population[7]. It contains 3 dimensions like objective support(3 items), subjective support(4 items) and availability of support(3 items) with a total score ranging from 7-56, the higher score, the higher level of your social support.

SSI, designed by Beck, is a splendid tool to sift out people with suicidal ideation and attempts[8]. 19 items of which could be divided into 2 parts. Part 1, which contains 5 items, were used to determine to what extent suicidal ideation was. The rest 14 items, which constitutes part 2, to determine to what extent suicidal attempt was. A total

score combining 2 parts were used to evaluate risk of suicide, which would range from 0-100. Both tick "no" in item 4 and 5 means no ideation was exposed and SSI terminated. The higher score, the more risk exposed.

SF-12, simplified from SF-36 in 1995 with the purpose of saving time[9], was used for evaluations of quality of life. Most of the core items were retained and several minutes would be enough to finish this inventory. The higher score, the higher quality of life.

References:

[1]. Needham, D.M., et al., Core Outcome Measures for Clinical Research in Acute Respiratory Failure Survivors. An International Modified Delphi Consensus Study. Am J Respir Crit Care Med, 2017. 196(9): p. 1122-1130.

[2]. Zhang, J., et al., [Relationship between social support, resilience, self-esteem and post-traumatic stress disorder in intensive care unit nurses]. Zhonghua Yi Xue Za Zhi, 2020. 100(1): p. 32-36.

[3]. Yue, T., et al., Comparison of Hospital Anxiety and Depression Scale (HADS) and Zung Self-Rating Anxiety/Depression Scale (SAS/SDS) in Evaluating Anxiety and Depression in Patients with Psoriatic Arthritis. Dermatology, 2020. 236(2): p. 170-178.

[4]. Foa, E.B., et al., The Obsessive-Compulsive Inventory: development and validation of a short version. Psychol Assess, 2002. 14(4): p. 485-96.

[5]. Gong, H., et al., The Obsessive-Compulsive Inventory-Revised: Replication of the psychometric properties in China. Bull Menninger Clin, 2020. 84(Supplement A): p. 34-47.

[6]. Xiao, H., et al., The Effects of Social Support on Sleep Quality of Medical Staff Treating Patients with Coronavirus Disease 2019 (COVID-19) in January and February 2020 in China. Med Sci Monit, 2020. 26: p. e923549.

[7]. SY., X., Theoretical foundation and research application about the social support rating scale. J Clin Psychiatry., 1994(4): p. 98－100.

[8]. Beck, A.T., M. Kovacs and A. Weissman, Assessment of suicidal intention: the Scale for Suicide Ideation. J Consult Clin Psychol, 1979. 47(2): p. 343-52.

[9]. Gandek, B., et al., Cross-validation of item selection and scoring for the SF-12 Health Survey in nine countries: results from the IQOLA Project. International Quality of Life Assessment. J Clin Epidemiol, 1998. 51(11): p. 1171-8.

校对报告

当前使用的样式是 [Numbered(Multilingual)]
当前文档包含的题录共10条
有2条题录存在必填字段内容缺失的问题
参考文献 [6] ：字段(期)内容缺失;
参考文献 [7] ：字段(卷)内容缺失;
