# Supplementary figures and images for: Possible Vicarious Traumatization Among Psychiatric Inpatients During the Remission Phase of the COVID-19: A Single-Center Cross-Sectional Study
Source: Front Psychiatry. 2021 Aug 24;12:677082. doi: 10.3389/fpsyt.2021.677082 (PMC8421644; doi:10.3389/fpsyt.2021.677082)

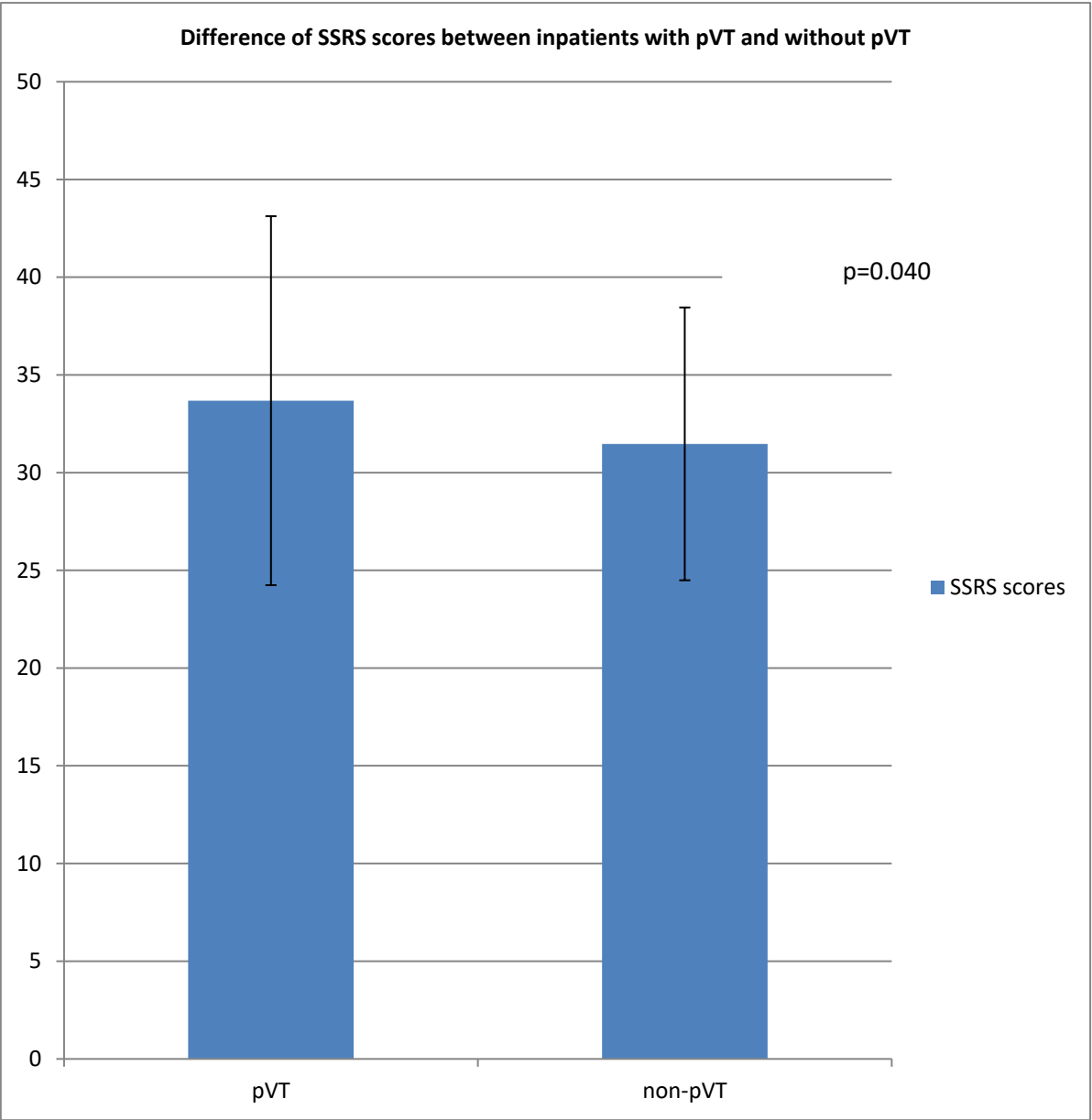

Supplement: Supplementary file 4 [file Data_Sheet_4.pdf]
